# *TERT* Mutation Is Accompanied by Neutrophil Infiltration and Contributes to Poor Survival in Isocitrate Dehydrogenase Wild-Type Glioma

**DOI:** 10.3389/fcell.2021.654407

**Published:** 2021-04-30

**Authors:** Mengqi Gao, Yi Lin, Xing Liu, Zheng Zhao, Zhiyuan Zhu, Hongbo Zhang, Yunchao Ban, Yanan Bie, Xiaozheng He, Xiang Sun, Shizhong Zhang

**Affiliations:** ^1^Guangdong Provincial Key Laboratory on Brain Function Repair and Regeneration, Department of Functional Neurosurgery, Zhujiang Hospital of Southern Medical University, Guangzhou, China; ^2^Department of Neurosurgery, The First Hospital of China Medical University, Shenyang, China; ^3^Chinese Glioma Genome Atlas Network, Beijing, China; ^4^Department of Neurosurgery, Beijing Tiantan Hospital, Capital Medical University, Beijing, China; ^5^Division of Neurosurgery, Department of Surgery, Li Ka Shing Faculty of Medicine, The University of Hong Kong, Hong Kong, China; ^6^School of Life Sciences and Biopharmaceutics, Guangdong Pharmaceutical University, Guangzhou, China

**Keywords:** *TERT* mutation, glioma, IDH mutation, neutrophil, chemokines, RNA-seq

## Abstract

Mutation of the telomerase reverse transcriptase (TERT) promoter has been demonstrated as an unfavorable prognostic marker in patients with isocitrate dehydrogenase wild-type (IDHwt) glioma. This study aimed to investigate the immune role of *TERT* promoter mutation status which could improve prognostic prediction in IDHwt. *TERT* mutation status, *IDH* mutation, and 1p-19q codeletion status data were obtained from 614 glioma cases from the Cancer Genome Atlas, and 325 cases from the Chinese Glioma Genome Atlas. The same information was obtained from 49 clinical glioma tissues. *TERT* mutation is preferentially present in glioblastoma and IDH-wt gliomas and is associated with poor prognosis. Moreover, *TERT* mutation was associated with infiltration of neutrophils and expression of neutrophil chemokines. which might partially contribute to the poor outcome in IDH-wt glioma. Furthermore, patients with IDH-wt glioma did not harbor increased peripheral neutrophils, implying that the infiltrated neutrophil in the tumor environment might due to cytokine chemotaxis. In this study, we hereby propose that TERT mutation might be a molecular driver of the dysfunctional immune microenvironment in IDH-wt glioma. *TERT* mutation may be a potential immune therapeutic target for optimizing treatment combinations and patient selection for glioma immunotherapy.

## Introduction

Glioma is the most common and malignant primary brain tumor in adults (Weller et al., 2015). Telomerase re-activation is frequent in malignancies and confers replicative immortality. It has been associated with poor outcome in brain tumors (Lotsch et al., 2013; Gojo et al., 2017; Gabler et al., 2019). Telomerase, the key enzyme for telomere length maintenance, is crucial for the survival of cancer cells (Duan and Zhao, 2018). Through its activation, tumor cells circumvent cellular senescence caused by telomere shortening, thus obtaining immortality (Martinez and Blasco, 2011). In brain cancers, concurrent mutations of *BRAF* and *TERT* promoter have been identified to be associated with tumor aggressiveness (Mistry et al., 2015; Matsumura et al., 2017; Mackay et al., 2018; Phillips et al., 2019). Telomeres are regions of repetitive nucleotide sequences (TTAGGG). They are composed of a DNA and telomere-binding proteins that at the ends of chromosomes (Blackburn et al., 2015). Ohali et al. (2006) reported that telomere length is associated with neuroblastoma prognosis. It is a reverse transcriptase that is composed of a catalytic protein subunit called telomerase reverse transcriptase (TERT), coding by the TERT gene in humans, and a human telomerase RNA (hTR) element encoded by hTERC (Duan and Zhao, 2018). Previous research have shown that *TERT* downregulation obviously inhibits the proliferation and invasion of neuroblastoma cells, and induces apoptosis (Chakrabarti et al., 2013). The poor prognosis of glioma is result from the transformed cells combining the tumor microenvironment, including vascular and stromal cells and inflammatory infiltrates (Hewedi et al., 2013; Poon et al., 2017). Current glioma diagnosis and classification system scarcely interrogates the effect of *TERT* mutation on glioma immune microenvironment. Here, we aimed to identify biomarkers associated with *TERT* promoter mutation status to improve prognosis prediction in IDH-wt glioma.

## Materials and Methods

### Patients and Databases

Datasets of The Cancer Genome Atlas (TCGA)^1^ and the Chinese Glioma Genome Atlas (CGGA)^2^ datasets were downloaded online. This study collected transcriptome expression data of 614 TCGA gliomas and 325 CGGA gliomas, including age, gender, diagnosis, WHO classification, chemotherapy and radiotherapy regimens, molecular data and prognosis. Meanwhile, tumor tissues of 49 gliomas who received surgical treatment in the first hospital of China Medical University from 2017 to 2018 were collected, including 12 WHO grade II, 22 WHO grade III and 15 glioblastoma patients (Supplementary Table 1). This study was approved by the ethics committee of the First Hospital of China Medical University. Written informed consent was obtained from all patients.

### TERT Mutation

The *TERT* mutation status and mRNA expression data were downloaded from TCGA. The *TERT* status of 49 glioma patients was determined by sequencing.

### *IDH* Mutation and 1p-19q Codeletion Status

*IDH* mutation and 1p-19q codeletion status data were downloaded from the TCGA and CGGA datasets. The IDH1 and IDH2 status of 49 glioma patients was ascertained as reported earlier (Gao et al., 2019).

### Immunohistochemistry and Immunofluorescence

Formalin fixed paraffin embedded tissues were used for immunohistochemistry and immunofluorescence. Four micron thick slices were cut, dewaxed in xylene, rinsed in graded ethanol, and rehydrated in distilled water. After the antigen was extracted with sodium citrate buffer (10 mM sodium citrate, pH 6.0), the endogenous peroxidase activity was blocked by 3% H_2_O_2_. The sections were incubated with primary antibodies and DAB staining solution was used for signal generating. Sections were stained with hematoxylin, dehydrated and sealed. For immunofluorescence, the antigen was extracted by EDTA buffer (1 mm Tris/EDTA, pH 9.0). The endogenous fluorescence was eliminated by AutoFluo Quencher (Servicebio, Wuhan, Cat# G1221). The details of the immunofluorescence and immunohistochemistry antibodies are showed in Supplementary Table 2. Image Pro Plus (v.6.0) was used to score the staining of each marker. The integral optical density area ratio of each marker was calculated to evaluate the staining intensity.

### Statistical Analysis

R language (v. 3.6.2), SPSS software (v. 22.0), and GraphPad Prism (v. 8.3.0) for Windows were used for statistical analyses and generating figures. Two-tailed Student’s *t*-test was used to separate the genes with differential expression according to TERT mutation. Correction of *p*-values was performed to control the false discovery rate (FDR) using *R*-values of FDR < 0.01, which were considered statistically significant. The multivariate Cox proportional hazard model was used to evaluate independent prognostic variables, and Kaplan–Meier curves were employed to depict survival distributions. Immune cells that correlated with the *TERT* mutation status were explored by a two-tailed Student’s *t*-test using SPSS, considering the effect of variant grades or IDH status.

### Bioinformatics Analyses

The Database for Annotation, Visualization, and Integrated Discovery (DAVID v6.8)^3^, STRING (v10.5)^4^, and Kyoto Encyclopedia of Genes and Genomes (KEGG)^5^ were performed to gene annotation and pathway analyses. Gene set enrichment analysis (GSEA) was used to explore the biological functions of *TERT* mutations. To further define the immune cell subpopulations affected by *TERT* mutation, we used gene set variation analysis (GSVA) to explore the relationship between TERT and the predefined, highly distinctive, transcriptional profile of each immune cell type (Chtanova et al., 2005; Wendt et al., 2006; Hyrcza et al., 2007; Bindea et al., 2013). Eighteen types of immune cells with corresponding gene signatures were selected (Supplementary Table 3).

## Results

### TERT Mutation Tended to Occur Frequently in Glioblastoma and IDHwt Glioma, With a Trend for Poor Survival

**FIGURE 1:**
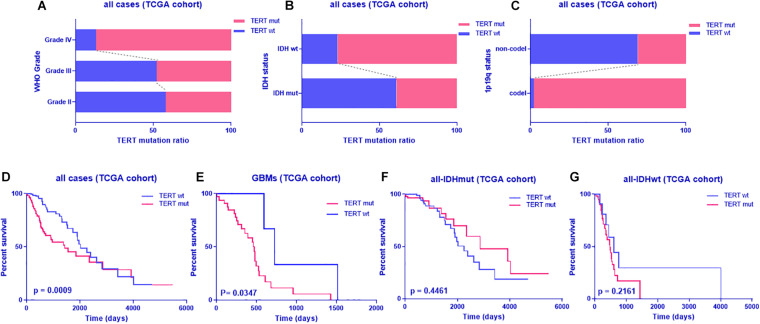
TERT mutation pattern and prognostic value in glioma from TCGA dataset. TERT mutation radio in glioma according to WHO grade **(A)**, IDH status **(B)** or 1p-19q co-deletion **(C)**; Kaplan-Meier analyses of overall survival according to TERT status (**D**, all cases; **E**, glioblastoma; **F**, all cases with IDH mutant; **G**, all cases with IDH wild type); discriminative power of TERT was assessed with Kaplan-Meier plotting method and the Log-rank test. Significant *p* < 0.05.

To identify the distribution of *TERT* status, we analyzed the *TERT* mutation ratio by hierarchical analysis. First, we found that glioblastomas are more prone to *TERT* mutations than grade II and grade III gliomas (Figure 1A) as well as gliomas with IDHwt or 1p19q codeletion (Figures 1B,C). Furthermore, *TERT* mutant (*TERT*mut) gliomas were associated with shorter patient survival across glioblastoma and IDHwt subgroups, despite no significant difference from IDHwt subgroups (Figures 1D–G and Supplementary Table 4).

### TERT Mutation Is Associated With Immune Response Promotion in IDH-wt Glioma

**FIGURE 2:**
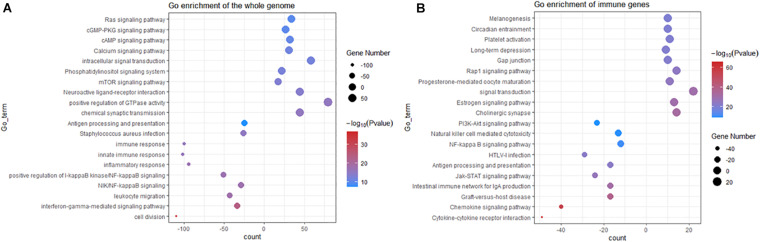
GO analyses of upregulated gene set enrichment according to *TERT* mutation in IDHwt gliomas (including KEGG). **(A)** GO analyses enrichment pathways for differentially expressed genes from the whole RNA-Seq genes among IDHwt gliomas. **(B)** GO pathway analyses for differentially expressed immune genes among IDHwt gliomas; Plot size shows gene counts on enrichment of pathway; Color depth shows the –log10p value from low level (blue) to high (red); significant *p* < 0.05.

To better understand the mechanism of *TERT* mutation in IDHwt glioma, we determined the differentially expressed genes between *TERT* wild-type and mutation. Among the 20,497 genes analyzed, 3,265 genes were upregulated in *TERT*mut gliomas, and 1,682 genes showed increased expression in *TERT* wild-type ones (adjusted *p* < 0.01, Supplementary Table 5). Subsequent analyses showed that the upregulated genes in *TERT*mut gliomas were enriched in various immune-related pathways including cell division, interferon-gamma-mediated signaling pathway, NF-κB signaling pathway, positive regulation of NF-κB signaling, inflammatory response, innate immune response, immune response, and antigen processing and presentation (Figure 2A). Therefore, we considered that the *TERT* promoter status was associated with the immune response in glioma. Among the 822 immune genes, the expression levels of 361 genes were upregulated in *TERT*mut gliomas and 91 genes in *TERT* wild-type cells (*t*-test, *p* < 0.05, Supplementary Table 5). We then analyzed the *TERT* mutation-related immune genes using Gene Ontology (GO). GO analyses showed that multiple immune-related processes were enriched in *TERT*mut gliomas, including cytokine-cytokine receptor interaction, chemokine signaling pathway, graft-versus-host disease, intestinal immune network for IgA production, Jak-STAT signaling pathway, antigen processing and presentation, HTLV-I infection, NF-κB signaling pathway, natural killer cell-mediated cytotoxicity, and PI3K-Akt signaling pathway (Figure 2B), which verified our hypothesis that *TERT* promoter status is associated with immune response in glioma. We further validated this hypothesis using GSEA, which highlighted that *TERT*mut gliomas showed increased immune responses (Supplementary Figure 1).

### TERT Mutation Is Accompanied by Increased Neutrophils in the Local IDH-wt Glioma Microenvironment

**FIGURE 3:**
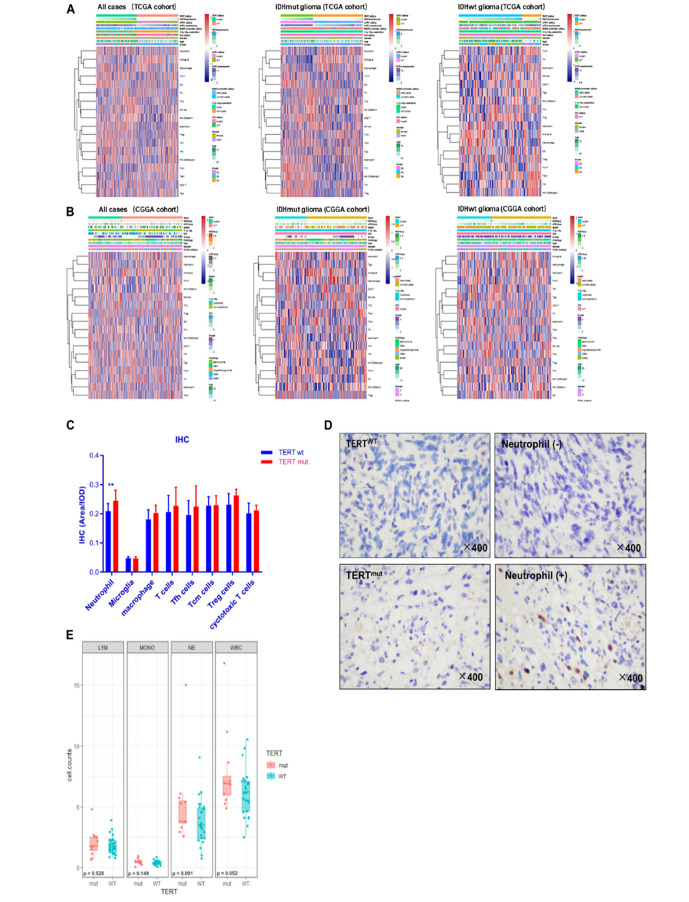
Immune cell enrichment in gliomas according to TERT status. Samples were divided into a *TERT* mutant (*TERT*mut) group and *TERT* wild-type (*TERT*wt) group. All cases, IDH mutant and IDH wild-type cases from TCGA cohort **(A)**. The same subgroup gliomas from the Chinese Glioma Genome Atlas (CGGA) cohort **(B)**. Immunostaining results of multiple immune cells according to *TERT* mutation. **(C)** Immunohistochemistry result among 49 IDHwt gliomas. **(D)** Immunohisto- chemical staining showed that neutrophils increased with *TERT* mutation in IDHwt gliomas, –, Lower than the mean value; +, higher than the mean value. **(E)** Immune cell counts of peripheral blood according to *TERT* mutation in IDHwt glioma; LYM, lymphocytes; MONO, monocytes; NE, neutrophils; WBC, total leukocytes; significant *p* < 0.05.

To further stratify the immune variation of *TERT* mutated tumors in glioma microenvironment, we analyzed the enrichment scores of 26 immune cell-characterized gene sets across *TERT* mutation status (Table S6). We found that *TERT*mut glioma contained infiltration of neutrophils (r^TERTmut^ = 0.054 ± 0.361, r^TERTwt^ = −0.203 ± 0.286, *p* < 0.01, TCGA cohort, *t*-test) in IDHwt glioma cases, whereas the numbers of microglia and central memory T-cells (Tcm) were low (Figure 3A). The same trend of neutrophils was observed in the CGGA cohort (Figure 3B, r^TERTmut^ = 0.091 ± 0.364, r^TERTwt^ = −0.044 ± 0.332, p < 0.05, CGGA cohort, *t*-test). However, macrophages were not correlated with TERT status.

To confirm the above findings, we investigated tumoral infiltrated immune cell in 49 IDH-wt glioma tissue and interrogated their association with *TERT* mutation status (Figures 3C,D). In line with the bioinformatics findings, the profiles of infiltrated immune cells differed between *TERT*mut and *TERT* wild-type gliomas. More neutrophils were identified in *TERT*mut gliomas (*p* < 0.01).

We then measured the level of peripheral neutrophils in preoperative patients. We found insignificant difference in neutrophil counts between *TERT*mut and wild-type gliomas (Figure 3E). Therefore, we speculated that the neutrophil enrichment in the *TERT*mut glioma local microenvironment was due to cytokine-induced chemotaxis.

### TERT-Mut Is Accompanied by Increased Neutrophils Relative to Chemokines in the Tumor

**FIGURE 4:**
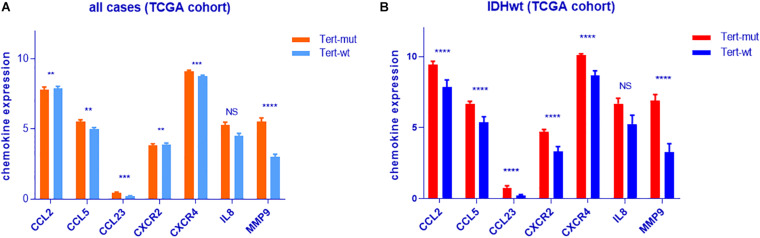
Analyses of neutrophil chemokine expression in IDHwt glioma according to *TERT* mutation. **(A)** All samples from TCGA cohort. **(B)** IDHwt samples from TCGA cohort. **p < 0.01; ***p < 0.001; ****p < 0.0001; NS, not significant.

To investigate the relationship between *TERT* mutation and neutrophil enrichment in glioma, chemokine levels were compared between *TERT*mut glioma and *TERT* wild-type glioma (Supplementary Table 7). We found that the following neutrophil chemokines—MMP9, CCL2, CCL5, CXCR4, CXCR2, CCL23, and IL8 (*p* = 0.07)—were enriched in *TERT*mut gliomas (*t*-test, *p* < 0.05, Figure 4).

### Neutrophil Enrichment in Tumor Microenvironment Is Related With the Survival of Glioma Patients

**FIGURE 5:**
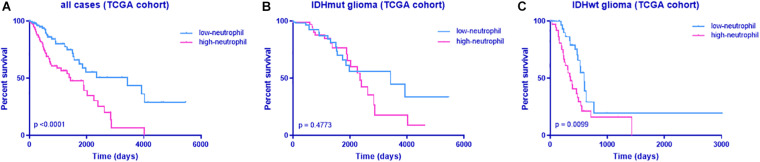
Kaplan–Meier analyses of overall survival according to the neutrophil enrichment score from GSVA. **(A)** All cases, **(B)** all cases with IDH mutant, and **(C)** all cases with IDH wild-type. The discriminative power of TERT was assessed with the Kaplan–Meier plotting method and the log-rank test. Significant *p* < 0.05.

To identify whether neutrophil infiltration accounts for poor survival of patients with *TERT*mut glioma, we analyzed the prognosis in cases based on neutrophil enrichment scores from the GSVA results. As expected, high neutrophil levels indicated poor survival in patients with glioma (Figure 5). This observation was in accordance with the findings in *TERT* mutation.

## Discussion

Inflammation promotes the proliferation, survival and metastasis of tumor cells, which is helpful to overturn adaptive immunity and enhance response to chemotherapy (Mantovani et al., 2008). The microenvironment of glioma is dominated by macrophages, which are thought to be transformed by glioma cells to promote tumor growth (Morantz et al., 1979; Poon et al., 2017). The poor survival of IDHwt gliomas may, at least partially, result from high levels of immune components infiltration (Yu et al., 2010). However, the underlying molecular mechanisms is still unclear. Therefore, more relevant studies are needed to provide deeper insights for improving precisive diagnosis and therapy for glioma (Roth et al., 1998; Rohn et al., 2001; Brown et al., 2017; Yang et al., 2017).

To this end, we proposed that the *TERT* mutation may play essential roles in mediating IDH-related immune response in tumor microenvironment (TME). We found that *TERT* mutation is associated with neutrophil enrichment, indicating that this mutation may closely associated with tumor immunity. Moreover, we identified a variety of specific chemokines infiltrated in *TERT*mut tumors, which might attribute to the chemotaxis of neutrophils in the microenvironment.

Tumor-associated neutrophils (TANs) release neutrophil elastase (ELA2), collagenase (MMP8) and gelatinase B (MMP9), which contain in their granules. These enzymes can promote the invasion of tumor cells by remodeling the extracellular matrix or directly acting on tumor cells (Dumitru et al., 2013; Bonavita et al., 2016). Neutrophils can even enhance the production of VEGF and the invasion of tumor cells by producing oncostatin M (Nozawa et al., 2006) and MMP9. On the other hand, neutrophils can inhibit tumor growth through antibody-dependent cytotoxicity (Stoppacciaro et al., 1993), which determines its important role in anti-cancer monoclonal antibody therapy (Brandsma et al., 2015). Interferon (IFN)-activated neutrophils can release TRAIL/APO2 ligand (tumor necrosis factor-related apoptosis-inducing ligand), which selectively induces apoptosis in tumor cells (Kemp et al., 2005; Cassatella et al., 2006). TANs may be classified into IFN-β-induced anti-tumoral neutrophils (N1), and TGF-β- induced tumor-promoting neutrophils (N2). N1 and N2 can be distinguished by various biological functions, such as expression of adhesion molecules, inflammatory mediators, chemokines, and chemokine receptors (Jablonska et al., 2010; Eruslanov et al., 2014).

These studies show that tumor-infiltrating neutrophils can be polarized. However, it is still unclear whether the functional differences of different neutrophils states is due to the regulation of cytokines in tumor microenvironment or the infiltration of different neutrophil subsets. CCRL2/CXCR2 is the main chemokine of neutrophils *in vitro* (Del Prete et al., 2017), which regulate both adaptive and innate immune responses (Del Prete et al., 2013). Blocking CXCR2 receptor can inhibit neutrophil infiltration into tumor, suppress tumor growth and reduce angiogenesis (Jablonska et al., 2014). In breast cancer, inhibition of CXCR2 increases the efficacy of chemotherapy (Acharyya et al., 2012). These data suggest that CXCR2 expressed by neutrophils is essential for their homing to tumors tissue with high expression of CXCR2 ligand. In tumors, CXCR4 is overexpressed in circulating neutrophil subsets, which promotes angiogenesis and tumor progression (Massena et al., 2015). Furthermore, CXCR4 may elevated in N2 neutrophils since it is inhibited by IFN-β (Jablonska et al., 2010). The expression of the CC chemokine receptors CCR1, CCR2, CCR3, and CCR5 is upregulated by neutrophils (Hartl et al., 2008). There have been emerging studies on the role of CCL2-CCR2 axis by neutrophil-monocyte cooperation biology (Hagerling et al., 2019; Hou et al., 2020). In tumor, neutrophils can be mobilized and recruited through CCL2–CCR2 axis (Pahler et al., 2008). However, whether CCL2 has positive or negative effects on tumor growth depends on it recruits pro-tumor or anti-tumor neutrophils/monocytes to the tumor (Mitchem and DeNardo, 2012; Mitchem et al., 2013; Lavender et al., 2017).

In this study, CCL2, CCL5, CXCR4, MMP9, and CXCR2 expression was found to be high in *TERT*mut glioma from all samples and IDHwt subgroups with high levels of neutrophil infiltration, which indicated that N2 phenotype neutrophils were associated with *TERT* mutation (Bonavita et al., 2016). In summary, overexpression of these chemokines may recruit specific neutrophils into the local tumor microenvironment and exhibiting anti-glioma effects.

The relationship between *TERT*mut and patient survival did not reach statistical significance in IDH subgroups. It could be result from the limited number of cases in each subgroup. Further studies are needed to determine the prognostic value of *TERT* mutations in these subgroups of patients. Further studies are needed to validate the relationship between Tert mutation and tumor associated neutrophil. *Ex vivo* profiling of the cell components in IDHwt glioma tissues would provide valuable information of tumor microenvironment. The correlation analysis can only provide preliminary evidence of the relationship rather than determine the causal relationship between Tert mutation and tumor associated neutrophil infiltration.

To conclude, this study suggest that *TERT* correlates with immune response and the infiltration of neutrophils in the IDH wild-type glioma microenvironment. Accordingly, *TERT* may serve as a potential therapeutic target. Further studies are warranted to confirm our findings and unveil the underlying mechanism.

## Data Availability Statement

The original contributions presented in the study are included in the article/Supplementary Material, further inquiries can be directed to the corresponding author/s.

## Ethics Statement

This study was approved by the Ethics Committee of The First Hospital of China Medical University. Written informed consent was obtained from all patients.

## Author Contributions

MG performed the study and drafted the manuscript. YL performed the study and revised the manuscript. YL and SZ supervised the study and revised the manuscript. All authors have read and approved the final manuscript.

## Conflict of Interest

The authors declare that the research was conducted in the absence of any commercial or financial relationships that could be construed as a potential conflict of interest.

## Footnotes

1 http://cancergenome.nih.gov

2 http://www.cgga.org.cn

3 http://david.abcc.n-cifcrf.gov

4 https://string-db.org/

5 http://www.kegg.jp/kegg/pathway.html

## Supplementary Material

Supplementary Figure 1 GSEA analyses of upregulated gene set enrichment in IDHwt glioma according to TERT mutation. **(A)** GO gene set enrichment in *TERT*mut glioma. **(B)** GO gene set enrichment in *TERT*wt glioma. **(C)** Immunologic gene set enrichment in *TERT*mut glioma. **(D)** Immunologic gene set enrichment in *TERT*wt glioma. Blue column with upper scale, NES; gray plot with lower scale, −log10 *p*-value; *p* < 0.05.
