# Supplementary figures and images for: TERT Mutation Is Accompanied by Neutrophil Infiltration and Contributes to Poor Survival in Isocitrate Dehydrogenase Wild-Type Glioma
Source: Front Cell Dev Biol. 2021 Apr 30;9:654407. doi: 10.3389/fcell.2021.654407 (PMC8119999; doi:10.3389/fcell.2021.654407)

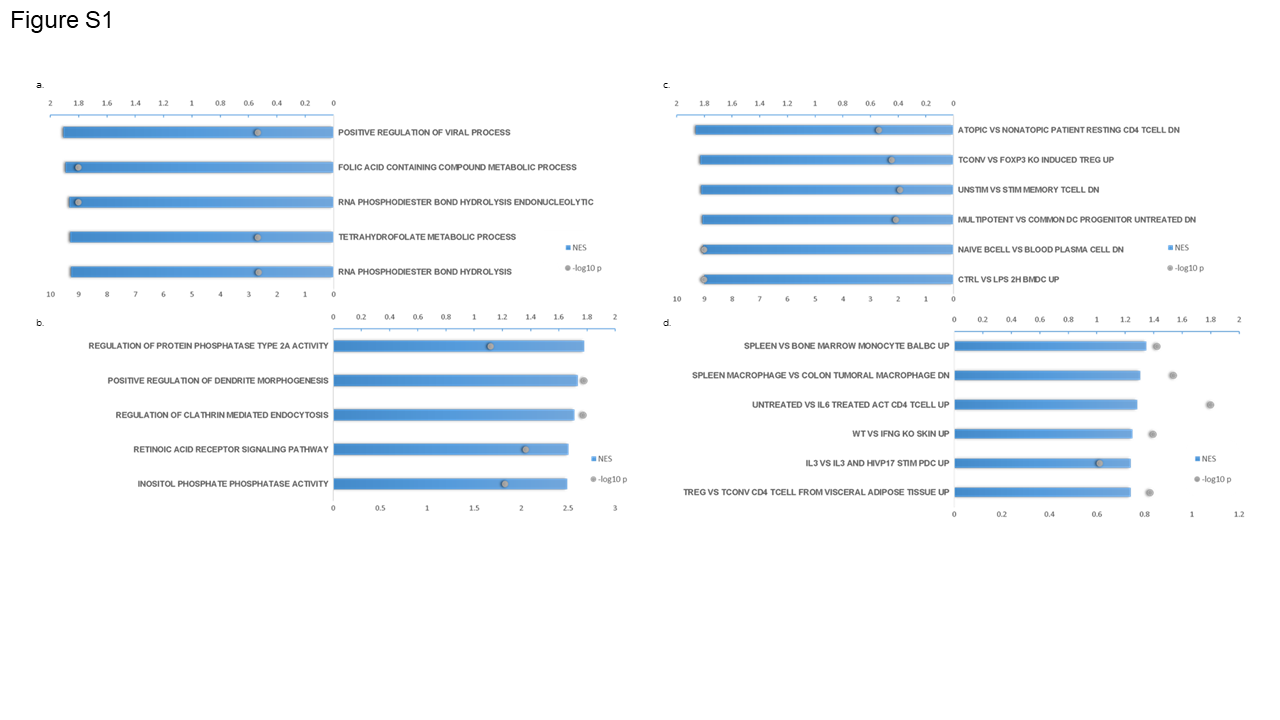

Supplement: Supplementary Figure 1 — GSEA analyses of upregulated gene set enrichment in IDHwt glioma according to TERT mutation. (A) GO gene set enrichment in TERTmut glioma. (B) GO gene set enrichment in TERTwt glioma. (C) Immunologic gene set enrichment in TERTmut glioma. (D) Immunologic gene set enrichment in TERTwt glioma. Blue column with upper scale, NES; gray plot with lower scale, −log10 p-value; p < 0.05. [file Image_1.tif]
