# Supplementary material for: TERT Mutation Is Accompanied by Neutrophil Infiltration and Contributes to Poor Survival in Isocitrate Dehydrogenase Wild-Type Glioma
Source: Front Cell Dev Biol. 2021 Apr 30;9:654407. doi: 10.3389/fcell.2021.654407 (PMC8119999; doi:10.3389/fcell.2021.654407)
Supplement: Supplementary file 5 [file Table_4.DOCX]

Correlation of Tert mutation and common clinicopathological factors with patients’ overall survival in the CGGA and TCGA datasets

| **Variables** | **Univariate analysis** | | | **Multivariate analysis** | | |
| --- | --- | --- | --- | --- | --- | --- |
|  | **HR** | **95%CI** | **P value** | **HR** | **95%CI** | **P value** |
| ***TCGA cohort*** |  |  |  |  |  |  |
| **Tert mutant** | **2.124** | **1.348-3.347** | **0.001*** | **2.316** | **1.103-4.863** | **0.026*** |
| Grade | 4.855 | 3.784-6.229 | 0.000* | 2.147 | 1.401-3.290 | 0.000* |
| IDH mutant | 0.096 | 0.068-0.137 | 0.000* | 0.306 | 0.141-0.663 | 0.003* |
| 1p19q co-deletion | 0.222 | 0.131-0.378 | 0.000* | 0.387 | 0.145-1.034 | 0.058 |
| MGMT methylation | 0.325 | 0.233-0.451 | 0.000* | 1.132 | 0.600-2.133 | 0.702 |
| ***CGGA cohort*** |  |  |  |  |  |  |
| **Tert mutant** | **0.639** | **0.419-0.975** | **0.038*** | **0.816** | ***0.525-1.270*** | ***0.368*** |
| Grade | 3.477 | 2.716-4.452 | 0.000* | 2.604 | 1.913-3.545 | 0.000* |
| IDH mutant | 0.228 | 0.158-0.329 | 0.000* | 1.121 | 0.508-2.477 | 0.777 |
| 1p19q co-deletion | 0.135 | 0.068-0.267 | 0.000* | 0.324 | 0.134-0.784 | 0.012* |
| MGMT methylation | 0.526 | 0.371-0.745 | 0.000* | 0.607 | 0.404-0.912 | 0.016* |

HR: hazard ration, CI: confidence interval. *p<0.05.
